# Fumaric Acid Esters Do Not Reduce Inflammatory NF-κB/p65 Nuclear Translocation, ICAM-1 Expression and T-Cell Adhesiveness of Human Brain Microvascular Endothelial Cells

**DOI:** 10.3390/ijms160819086

**Published:** 2015-08-13

**Authors:** Axel Haarmann, Mathias Nehen, Annika Deiß, Mathias Buttmann

**Affiliations:** Department of Neurology, University of Würzburg, Josef-Schneider-Str. 11, Würzburg 97080, Germany; E-Mails: haarmann_a@ukw.de (A.H.); mathias.nehen@stud-mail.uni-wuerzburg.de (M.N.); deiss_a@ukw.de (A.D.)

**Keywords:** blood-brain barrier, endothelial cells, multiple sclerosis, dimethyl fumarate, monomethyl fumarate, cell adhesion, NF-κB, p38 mitogen-activated protein kinase

## Abstract

Dimethyl fumarate (DMF) is approved for disease-modifying treatment of patients with relapsing-remitting multiple sclerosis. Animal experiments suggested that part of its therapeutic effect is due to a reduction of T-cell infiltration of the central nervous system (CNS) by uncertain mechanisms. Here we evaluated whether DMF and its primary metabolite monomethyl fumarate (MMF) modulate pro-inflammatory intracellular signaling and T-cell adhesiveness of nonimmortalized single donor human brain microvascular endothelial cells at low passages. Neither DMF nor MMF at concentrations of 10 or 50 µM blocked the IL-1β-induced nuclear translocation of NF-κB/p65, whereas the higher concentration of DMF inhibited the nuclear entry of p65 in human umbilical vein endothelium cultured in parallel. DMF and MMF also did not alter the IL-1β-stimulated activation of p38 MAPK in brain endothelium. Furthermore, neither DMF nor MMF reduced the basal or IL-1β-inducible expression of ICAM-1. In accordance, both fumaric acid esters did not reduce the adhesion of activated Jurkat T cells to brain endothelium under basal or inflammatory conditions. Therefore, brain endothelial cells probably do not directly mediate a potential blocking effect of fumaric acid esters on the inflammatory infiltration of the CNS by T cells.

## 1. Introduction

Multiple sclerosis (MS) is a chronic degenerative autoimmune disease of the central nervous system (CNS). A disturbance of the blood-brain barrier (BBB) is central to its pathogenesis. The BBB is formed by highly specialized endothelial cells, controlling the exchange of solute, soluble factors and immune cells between blood and CNS tissue by complex molecular mechanisms [1]. A number of disease-modifying drugs approved for the treatment of patients with relapsing-remitting MS are supposed to reduce immune cell infiltration into the CNS. Natalizumab for example, a recombinant monoclonal IgG4 antibody against integrin α-4, inhibits mononuclear leukocyte adhesion to activated CNS endothelium [2]. In addition, it was recently found to protect the paracellular endothelial barrier function by blocking integrin α-4 expressed on CNS endothelial cells [3]. Another drug, the sphingosine-1-phosphate receptor (S1PR) modulator fingolimod, reduces T-cell infiltration into the CNS parenchyma by trapping T cells in peripheral lymphoid organs through functional antagonization of S1PR1 on lymphocytes [4]. In addition, it may decrease the extravasation of monocytes across CNS endothelial cells by activating endothelial S1PR5, thereby reducing endothelial NF-κB activation and inflammatory expression of adhesion molecules and chemokines [5].

Dimethyl fumarate (DMF), which is also approved for the disease-modifying treatment of patients with relapsing-remitting MS, is another drug that may reduce mononuclear immune cell infiltration of the CNS. Preventive treatment of C57BL/6 mice undergoing myelin oligodendrocyte glycoprotein (MOG)_35–55_-induced experimental autoimmune encephalitis (EAE), an animal model of MS, with DMF or even more so with its primary metabolite monomethyl fumarate (MMF) was found to reduce infiltration of the spinal cord by CD3-positive T cells and Mac-3-positive mononuclear cells when analyzed 27 days after EAE induction [6]. In contrast, the same group did not observe a DMF-mediated reduction of mononuclear immune cell extravasation in the spinal cord 41 and 74 days after EAE induction [7]. The mechanisms by which fumaric acid esters (FAE) reduce mononuclear immune cell infiltration of the CNS in an earlier phase of EAE and possibly also in patients with MS are not entirely clear.

Modes of action may include FAE effects on immune cells, such as an induction of T-cell apoptosis, resulting in peripheral blood lymphopenia [8,9], or a reduced adhesive and migratory capacity of T lymphocytes at the BBB [10,11]. In addition, FAE may theoretically target endothelial cells at the inflamed BBB and inhibit immune cell extravasation by direct effects on endothelial cells. In human umbilical vein endothelial cells (HUVEC), DMF but not MMF blocked the adhesion of leukocytes *in vitro* by downregulating the inflammatory-inducible expression of the endothelial adhesion molecules ICAM-1, VCAM-1 and E-selectin [12,13], possibly by inhibiting the nuclear entry of activated NF-κB/p65 resulting in reduced gene expression [14]. However, macrovascular HUVEC differ in many molecular and functional aspects from CNS microvascular endothelial cells [15]. Therefore, we set out to evaluate this potential mode of FAE action at the BBB in well characterized non-immortalized single donor human brain microvascular endothelial cells (HBMEC) at low passages.

## 2. Results

### 2.1. FAE Do Not Modulate NF-κB/p65 Nuclear Translocation or p38 MAPK Activation in HBMEC

**Figure 1:**
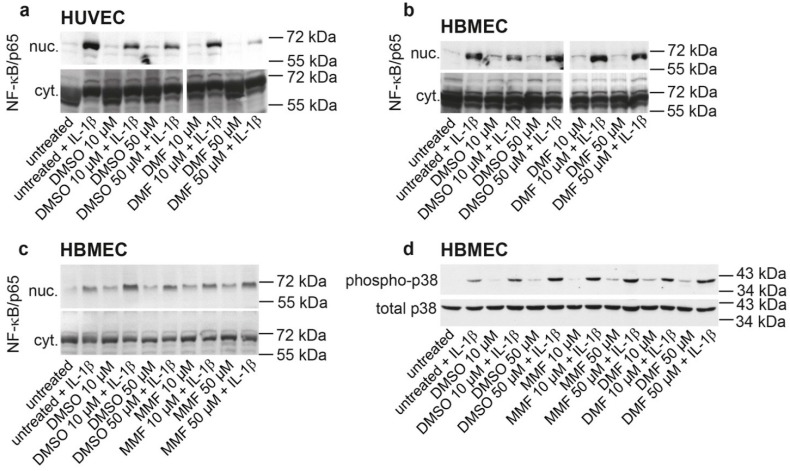
FAE do not modulate NF-κB nuclear translocation or p38 MAPK activation in HBMEC. HUVEC (**a**) or HBMEC (**b**) were cultured to subconfluency in parallel and then left untreated or exposed to 10 or 50 µM DMF, or to DMSO solvent control, corresponding to the DMSO concentrations present in the DMF samples, for 24 h. Subsequently, cells were either not additionally stimulated or 10 ng/mL IL-1β were additionally added for one further hour. NF-κB/p65 was then detected by Western blotting in nuclear (nuc.) and cytosolic (cyt.) cell protein extracts to investigate the nuclear translocation of NF-κB/p65; (**c**) experiment in HBMEC as in (**b**), however using MMF instead of DMF; and (**d**) treatment of HBMEC in analogy to (**b**,**c**) as indicated, followed by Western blotting against phosphorylated and total p38 MAPK. Each panel representative of three independent experiments.

To comparatively evaluate effects of FAE on the inflammatory-stimulated nuclear translocation of NF-κB/p65 in HUVEC *versus* HBMEC, both cell types were cultured in parallel to subconfluency. Subsequently, cells were pretreated with DMF, MMF or DMSO solvent control for 24 h and then additionally stimulated with IL-1β for 1 h or left without further stimulation. In both HUVEC and HBMEC, IL-1β induced a comparable nuclear translocation of NF-κB/p65. However, while in HUVEC DMF at the higher tested concentration of 50 µM clearly inhibited the nuclear entry of NF-κB/p65 (Figure 1a), which was in line with previously published results [14], neither DMF (Figure 1b) nor MMF (Figure 1c) blocked the nuclear entry of NF-κB in HBMEC compared to solvent control. Neither did DMF alter the basal or inflammatory activation of p38 MAPK in HBMEC (Figure 1d).

### 2.2. FAE Do Not Reduce Basal or Inflammatory-Inducible Expression of ICAM-1 in HBMEC

**Figure 2:**
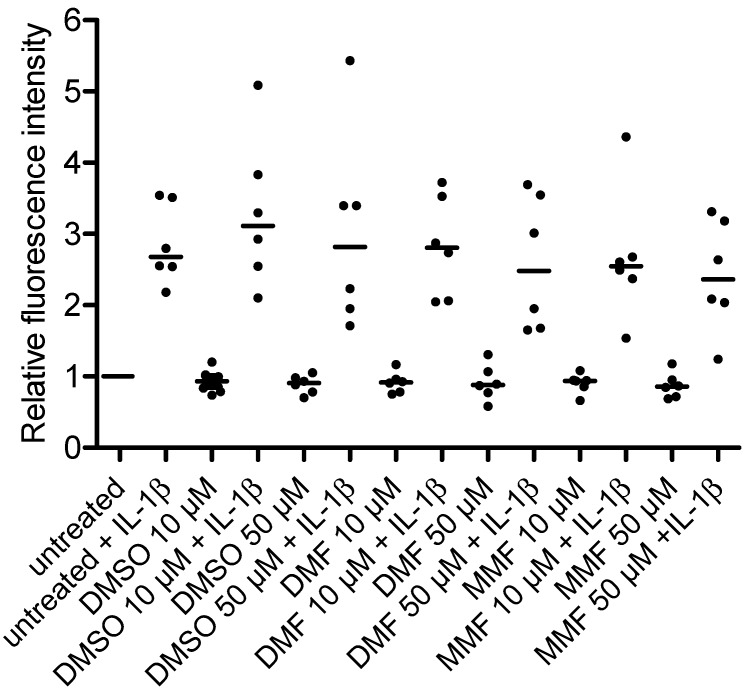
FAE do not reduce ICAM-1 expression on HBMEC under basal and inflammatory conditions. Subconfluent HBMEC were left untreated or exposed to DMF or MMF at concentrations of 10 or 50 µM, or to DMSO as a solvent control, at concentrations corresponding to the DMSO concentrations present in the FAE samples, for 24 h. Subsequently, cells were not additionally stimulated or 10 ng/mL IL-1β were additionally added for another 24 h. ICAM-1 surface expression after 48 h was assessed by flow cytometry. Geometric means of fluorescence intensities were compared to untreated cells and are reported as relative fluorescence intensities. Single values and medians of six independent experiments are shown.

Having observed that FAE did not reduce the IL-1β-inducible nuclear translocation of NF-κB/p65 nor did they modulate the activation of p38 MAPK in HBMEC, we next evaluated whether DMF or MMF alter the basal or inflammatory-inducible expression of ICAM-1 as a prototypic NF-κB-dependent adhesion molecule [16] with an important function for T-cell extravasation at the inflamed BBB [17]. Subconfluent HBMEC were pretreated with DMF, MMF or DMSO solvent control for 24 h and then additionally stimulated with IL-1β for another 24 h or did not receive additional stimulation. Neither DMF nor MMF changed the basal or inflammatory-inducible surface expression of ICAM-1 (Figure 2), which was in line with our previous result of a lack of NF-κB inhibition in HBMEC.

### 2.3. FAE Do Not Reduce T-Cell Adhesiveness of HBMEC

**Figure 3:**
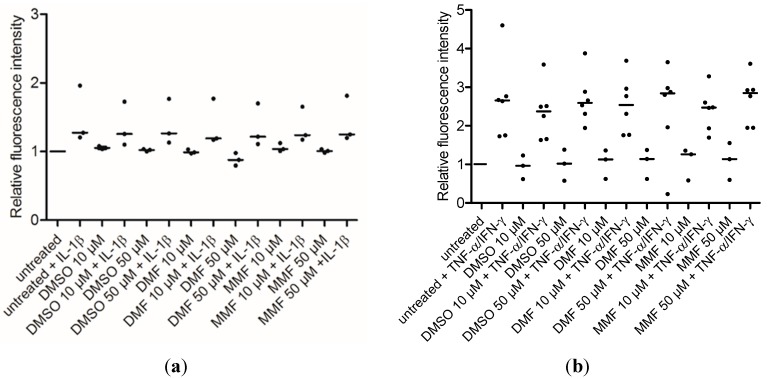
FAE do not reduce T-cell adhesiveness of HBMEC under basal and inflammatory conditions. Confluent HBMEC were stimulated in analogy to Figure 2, using IL-1β (**a**) or TNF-α plus IFN-γ (**b**) as inflammatory stimuli. Subsequently, adhesion of calcein-labeled, PMA-activated Jurkat T cells was assessed in a static adhesion assay. Adhesion of Jurkat T cells, corresponding to the measured fluorescence intensities, is shown relative to untreated cells. Dots represent means of single independent experiments in octuplicates, bars represent medians.

In a last step, we assessed whether FAE modulate the T-cell adhesiveness of HBMEC under resting or inflammatory conditions. Confluent HBMEC were pretreated with DMF, MMF or DMSO solvent control for 24 h and then additionally stimulated with IL-1β or with TNF-α plus IFN-γ for another 24 h or did not receive additional stimulation. Subsequently, adhesion of phorbol 12-myristate 13-acetate (PMA)-activated Jurkat T cells was assessed in a static adhesion assay. While inflammatory stimulation of HBMEC clearly increased their T-cell adhesiveness, neither DMF nor MMF reduced the adhesion of Jurkat T cells compared to solvent control under basal or inflammatory conditions (Figure 3).

## 3. Discussion

Here we report that FAE did neither reduce the inflammatory nuclear translocation of NF-κB/p65, nor the surface expression of ICAM-1, nor the T-cell adhesiveness of well characterized non-immortalized single donor HBMEC at early culture passages. This previously undescribed behavior of HBMEC is in contrast to findings in HUVEC, as independently reported by different groups and partially confirmed by ourselves in this study as a positive control [12,13,14]. Our *in vitro* results consistently argue that probably neither DMF, which after oral administration is not quantifiable in human plasma due to rapid hydrolysis [18] and therefore probably does not reach the human BBB at significant quantities, nor its immediate metabolite MMF directly act on endothelial cells at the inflamed BBB to reduce T-cell infiltration into the human CNS.

Our findings may be indirectly supported by an *in vivo* study that did not observe an antagonization of inflammatory claudin-5 downregulation at the BBB in DMF-treated C57BL/6 mice with MOG_35–55_-EAE, arguing for a lack of stabilization of another aspect of BBB function by FAE under inflammatory conditions. Using immortalized hCMEC/D3 cells for *in vitro* experiments, however, both DMF and even more so MMF antagonized the TNF-α-induced downregulation of claudin-5, occludin and zonula occludens-1 (ZO-1) mRNA and protein expression, which appeared to be in contrast to the *in vivo* results obtained by the same authors [19]. They concluded that their contradictory *in vitro* results may potentially be explained by the use of an immortalized cell line and that protection of the BBB is not a major mechanism *in vivo*. It should be noted, however, that in this study mice were analyzed for claudin-5 expression 72 days after EAE induction. The same authors had previously not observed an effect of DMF on CNS immune cell infiltration 41 and 74 days after MOG_35–55_-EAE induction [7], while reduced CNS immune cell infiltration was found 27 days after injection of MOG_35–55_ peptide [6]. It therefore seems conceivable that the chosen time point for analysis of claudin-5 expression was too late in the course of EAE to detect a protective effect of DMF treatment on BBB function. Such an effect still appears possible during earlier phases of the disease, which calls for additional investigations of the BBB during earlier phases of EAE.

A recent study exploring C57BL/6 mice undergoing experimental ischemia reperfusion injury, found a preservation of claudin-5 expression at the BBB along with reduced brain edema formation after oral pre-treatment of the mice with DMF [20]. Using the immortalized murine brain endothelial cell line bEND.3 for *in vitro* experiments, the same authors found a preservation of zonula occludens-1 (ZO-1) and VE-cadherin localization in oxygen-glucose deprived cells in the presence of DMF. Also employing bEND.3 cells, they observed reduced transendothelial migration of the human monocyte cell line THP-1 against CCL2 in the lower chamber of a transwell system after pre-treatment of the bEND.3 cells with DMF. They furthermore observed decreased ICAM-1, VCAM-1 and E-selectin mRNA expression in bEND.3 cells after treatment with 50 µM DMF for 6 h. In their transmigration assays and mRNA expression studies, bEND.3 cells were unstimulated (apart from the presence of CCL2), leaving open the effect of DMF under oxygen-glucose deprived conditions and mechanistically posing the question whether NF-κB was activated in the unstimulated cells. *In vitro* results for MMF, the presumed pharmacologically active FAE, were not reported. Despite the congruency of the reported *in vivo* results, the bEND.3 *in vitro* findings should be interpreted with caution in our view, as this cell line was criticized by a number of authors for a lack of typical BBB properties [21,22,23], which corresponds to our own experience with bEND.3 cells from various sources. In line with a limited barrier function of bEND.3 cells, the authors of the discussed study did not observe a protection of the paracellular barrier function by DMF [20].

To study the organ- and potentially also species-specific behavior of highly specialized endothelial cells at the human BBB, the preserved differentiation and human source of the used cells is of crucial importance in our view. The non-immortalized human cells at early passages employed in this study are well characterized and were successfully used by us for a number of studies in the past. These cells respond to inflammatory stimulation, as demonstrated here by NF-κB/p65 nuclear translocation, p38 MAPK activation as well as increased ICAM-1 expression and increased T-cell adhesiveness. Furthermore, we additionally demonstrated a successful modulation of functional BBB properties by different pharmacological agents in these cells in the past [3,24,25]. We, therefore, believe that the reported non-responsivity of our HBMEC to FAE probably reflects the true behavior of HBMEC at the human BBB *in vivo*. The use of immortalized, instead of primary, T cells for the adhesion assays represents a potential limitation of our study, although we successfully used them in a similar context in the past [25].

## 4. Materials and Methods

### 4.1. Cell Culture

Cryopreserved single donor primary HBMEC at passage 2 were purchased from Cell Systems Corp. (Kirkland, WA, USA) and cryopreserved single donor HUVEC at passage 1 from Promocell (Heidelberg, Germany). To exclude contamination by other cell types and to demonstrate the expression of tight junction-associated molecules, each HBMEC preparation was extensively characterized as previously described [3,24,25]. HBMEC purity was >98% in all preparations. Mycoplasma contamination was excluded using a commercial PCR-based mycoplasma detection kit (PK-CA91; PromoKine, Heidelberg, Germany). Cells were grown on 2% gelatin-coated plates or flasks (all from Nunc, Roskilde, Denmark) at 37 °C/5% CO_2_, using M199 basal medium (Lonza, Cologne, Germany) supplemented with 10% fetal calf serum (FCS; Biochrom, Berlin, Germany), endothelial cell growth supplement (20 μg/mL; Sigma-Aldrich, Schnelldorf, Germany), heparin (100 μg/mL; Sigma-Aldrich), amphotericin B (250 μg/mL), gentamycin (50 μg/mL), penicillin (50 U/mL) and streptomycin (50 μg/mL; all from Invitrogen, Karlsruhe, Germany). Endothelial cells were used between passage 3 and 6 for experiments. Jurkat T cells (clone E6-1) were purchased from ATCC (Manassas, VA, USA) and cultured in RPMI1640 medium (Gibco, Life Technologies GmbH, Darmstadt, Germany) supplemented by 10% FCS at 37 °C/5% CO_2_.

### 4.2. Stimulation

DMF and MMF were purchased from Sigma-Aldrich and dissolved in dimethyl sulfoxide (DMSO) to prepare 50 mM stock solutions which were stored in aliquots at −80 °C. For treatment of endothelial cells, FAE stock aliquots or DMSO as a solvent control were freshly diluted using M199 medium. Endothelial cells were treated with FAE at the indicated concentrations for the indicated durations. For inflammatory stimulation, endothelial cells were incubated with 10 ng/mL IL-1β, or with 10 ng/mL TNF-α and 100 IU/mL IFN-γ (all from R&D Systems, Karlsruhe, Germany) for the indicated durations. Jurkat T cells were activated by stimulation with 10 ng/mL PMA (Sigma-Aldrich) for 24 h.

### 4.3. Cell Protein Extracts and Western Blotting

For Western blot experiments, endothelial cells were grown to subconfluency and stimulated as indicated. Nuclear and cytosolic protein extracts were prepared exactly as previously described [26]. Cell protein extracts were fractionated by 10% SDS-PAGE and electroblotted onto nitrocellulose membrane. Ponceau staining served as a control for equal loading and complete protein transfer. Rabbit polyclonal primary antibodies (Abs) against NF-κB/p65 (1:500; Biotechnologies, Santa Cruz, CA, USA) or phospho-p38 (1:1000; Cell Signaling, Cambridge, UK) were incubated over night at 4 °C and detected with an anti-rabbit-IgG peroxidase-coupled secondary Ab along with a standard enhanced chemoluminescence system. After peroxidase inactivation, membranes for p38 detection were reprobed with mouse anti-p38 (clone A-12, 1:500; Biotechnologies, Santa Cruz, CA, USA) and an appropriate secondary Ab.

### 4.4. Flow Cytometry

HBMEC cultured on six-well plates to subconfluency were stimulated as indicated and subsequently detached with Accutase™ (PAA Laboratories, Coelbe, Germany). Then they were washed and incubated with a FITC-labeled mouse anti-ICAM-1 antibody (clone BBIG-I1, R&D Systems) or the corresponding FITC-labeled isotype control IgG1 (BD Biosciences, Heidelberg, Germany). Flow cytometry was performed on a FACSCalibur (BD Biosciences) fluorescence-activated cell sorter.

### 4.5. Adhesion Assay

Adhesion assays were performed exactly as previously described in detail [25]. Briefly, 5 × 10^4^ HBMEC per well were seeded on 96-well plates and grown to confluency which usually took 48 to 72 h. Subsequently, cells were incubated as indicated. After washing, calcein-labeled (Molecular Probes, Life Technologies), PMA-activated Jurkat T cells were added in FCS-free RPMI1640 medium for 30 min at 37 °C. After additional washing steps, fluorescence intensity, reflecting the number of adherent cells in a linear manner, was determined using a Fluoroskan Ascent microplate fluorometer (Thermo Sientific, Waltham, MA, USA).

## 5. Conclusions

Based on previously published experimental results, it seems conceivable that FAE reduce T-cell infiltration into the CNS parenchyma of patients with MS. Our findings, however, suggest that this potential mode of action, if present, is not mediated by a direct effect of FAE on endothelial cells at the BBB under inflammatory conditions.
